# Improved Wound Healing by Direct Cold Atmospheric Plasma Once or Twice a Week: A Randomized Controlled Trial on Chronic Venous Leg Ulcers

**DOI:** 10.1089/wound.2023.0196

**Published:** 2025-01-13

**Authors:** Olaf Bakker, Paulien Smits, Chantal van Weersch, Melissa Quaaden, Esther Bruls, Angela van Loon, Joost van der Kleij

**Affiliations:** ^1^ St. Antonius hospital, Nieuwegein, The Netherlands.; ^2^ Plasmacure B.V., Eindhoven, The Netherlands.; ^3^ Zuyderland Thuiszorg, Sittard, The Netherlands.; ^4^ Thebe Zorg Thuis, Tilburg, The Netherlands.; ^5^ Independent principal investigator, Maastricht, The Netherlands.

**Keywords:** cold plasma treatment, wound healing, venous leg ulcer, complex wounds, advanced therapy

## Abstract

**Objective:**

This study compared the effect of two frequencies of direct cold atmospheric plasma (direct-CAP) treatment with standard of care (SOC) alone on healing of venous leg ulcers (VLUs).

**Approach:**

Open-label, randomized controlled trial (ClinicalTrials.gov NCT04922463) on chronic VLUs at two home care organizations in the Netherlands. All three groups received SOC for 12 weeks or until healing. In addition, treatment groups received direct-CAP once (1× direct-CAP) or twice (2× direct-CAP) a week, at specialized wound care facilities and the patients’ residences. Primary outcome was percentage of wounds healed. Secondary outcomes included wound area reduction and adverse events.

**Results:**

In total, 46 patients were randomly allocated to receive SOC only (*n* = 15), SOC + direct-CAP once a week (*n* = 17), or SOC + direct-CAP twice a week (*n* = 14). A higher percentage of wounds healed within 12 weeks in the treatment groups 53.3% (1× direct-CAP, *p* = 0.16) and 61.5% (2× direct-CAP, *p* = 0.08) versus 25.0% (control). The largest wound area reduction was obtained with 2× direct-CAP (95.2%, *p* = 0.07), followed by 1× direct-CAP (63.9%, *p* = 0.58), versus control (52.8%). Absolute wound area reduced significantly compared with baseline in both treatment groups (*p* ≤ 0.001), not in control (*p* = 0.11). No device-related serious adverse events occurred.

**Innovation:**

Direct-CAP applied once or twice a week could substantially improve wound healing of VLUs in primary care.

**Conclusion:**

Together with other clinical safety and efficacy data, these results support the integration of direct-CAP as a valuable therapy for complex wounds.

**Figure:**
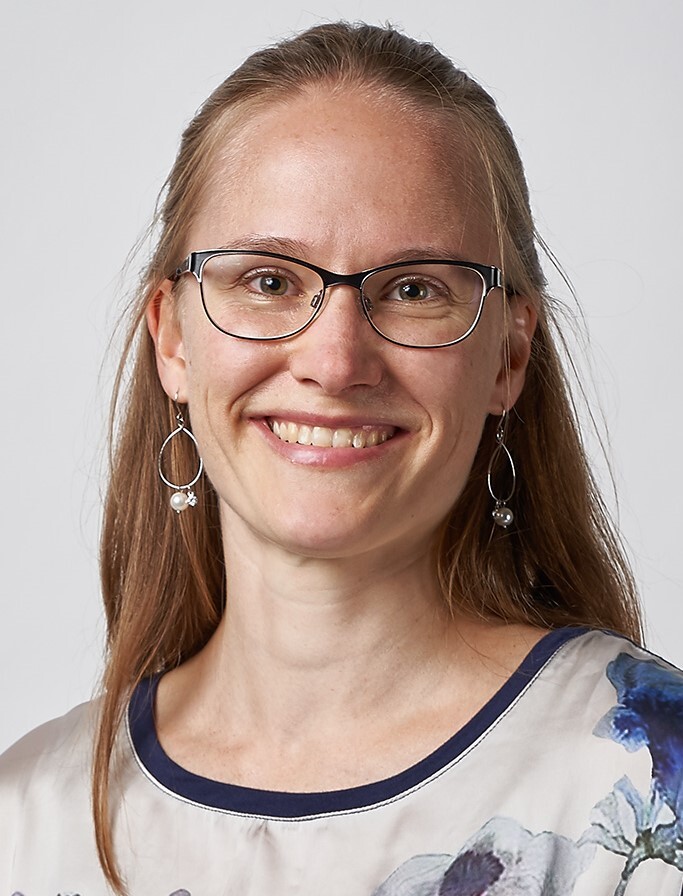
Paulien Smits

## INTRODUCTION

Complex or chronic wounds, such as venous leg ulcers (VLUs), present a multifaceted challenge within the health care sector. Such wounds do not progress through a normal and timely sequence of repair using conventional wound care and exert a profound impact on patients’ quality of life.^1–3^

**Figure 1.**
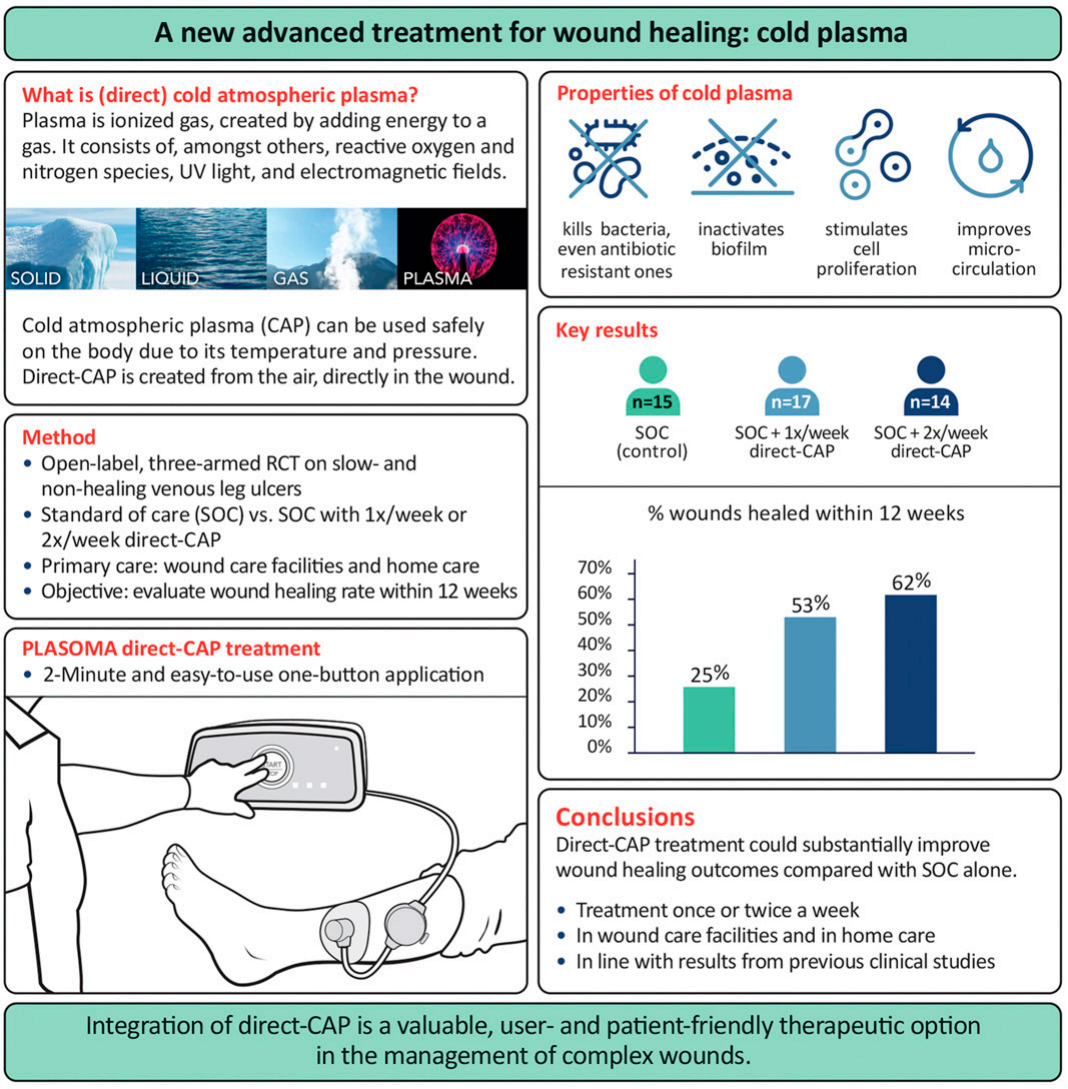
Summary containing an explanation of direct cold atmospheric plasma (direct-CAP) and its effects, a schematic diagram of the direct-CAP device, as well as highlights of the study methods, results, and conclusions. CAP, cold atmospheric plasma; RCT, randomized controlled trial; SOC, standard of care.

The underlying phenomena associated with the impaired healing of chronic wounds, irrespective of the latter’s origin, revolve around the predominant hypoxic and inflammatory environment, persistent infections, and the inability of skin cells to respond to reparative stimuli.^4^ Recently, cold atmospheric plasma (CAP) has shown potential in medical use, especially in wound care. Plasma is ionized gas, the fourth state of matter, created by adding energy to a gas. It consists of, among others, reactive oxygen and nitrogen species, UV light, and electromagnetic fields. CAP is plasma with a temperature not far above body temperature and at atmospheric pressure. CAP causes stimulating effects, including cell proliferation and microcirculation enhancement, as well as broad-spectrum microbial inactivation, even when antibiotic resistant and in biofilm (Fig. 1).^5,6^ These multiple modes of action of CAP are an advantage over other advanced wound care treatments.

This randomized controlled trial (RCT) aims primarily to assess the efficacy of direct cold atmospheric plasma (direct-CAP) treatment on the healing of slow-healing and nonhealing VLUs, specifically to evaluate the percentage of wounds healed after 12 weeks of treatment for two direct-CAP treatment frequencies plus standard of care (SOC) compared with SOC only. The direct-CAP device is Conformité Européene (CE) certified and prior clinical studies on complex wounds of various etiologies demonstrated promising results, including significant reductions in bacterial load and wound size in diabetic foot ulcers (DFUs).^7–9^

## INNOVATION

Despite the existence of numerous advanced treatments, a considerable number of complex wounds such as VLUs fail to heal. CAP, with its complex cocktail of reactive species and electric fields, is distinguished from other treatments by its multifaceted effects (Fig. 1). The application of direct-CAP can potentially heal a significant percentage of these hard-to-heal wounds with one treatment a week, which is easily integrated into the wound care routine.

## CLINICAL PROBLEM ADDRESSED

As our population ages and the number of comorbidities rises, the incidence of complex wounds, of which a considerable proportion remain unhealed with the current treatments, will increase. This leads to an escalating demand for care, and thus, innovative therapeutic approaches are urgently needed.^1,3^ While advanced treatment methods like advanced wound dressings, negative pressure therapy, and cell- or tissue-based therapies such as platelet-rich plasma exist, they often lack robust efficacy evidence or their applicability is limited due to costs, unavailability in home care, nonscalability, *etc.*^3,4,10^

CAP treatment is a relatively new advanced treatment, with ample preclinical and clinical evidence on the healing promoting effects in various complex wound types.^6^ Nevertheless, more high-quality RCTs are necessary to evaluate the efficacy of CAP on chronic wounds. Most CAP studies assess nonhealing outcomes, after a relatively short treatment period, instead of one of the most objective and clinically meaningful wound healing endpoints: incidence of complete wound closure.^11^ Furthermore, none of the existing RCTs has been executed outside health care facilities. In the current study, direct-CAP treatments were performed during a period of 12 weeks, also at the patient’s home, and the primary outcome was complete wound closure.

Another important feature of this study’s design is the incorporation of two treatment frequencies, once and twice per week. Previous clinical studies with other CAP devices each investigated a single treatment frequency (sequence). To our knowledge, there is only one RCT comparing the wound healing outcomes of two treatment frequencies, once and thrice per week.^12^ However, standard wound care is generally performed 1–2 times per week. Aligning the treatment frequency with the standard wound care regimen will facilitate integration of a new therapy.

## MATERIALS AND METHODS

### Study design

This multicenter, prospective, open-label, three-armed, parallel-group RCT was designed to examine further beneficial effects of direct-CAP treatment compared with SOC in patients with a chronic VLU (ClinicalTrials.gov Identifier: NCT04922463). The three study groups were: SOC only, SOC + once a week direct-CAP, SOC + twice a week direct-CAP.

The study was conducted in the Netherlands; the two participating study sites were home care organizations (primary care) with specialized wound care facilities: Zuyderland Thuiszorg (three locations: Kerkrade, Maastricht, Sittard) and Thebe Zorg Thuis, Tilburg.

The study complied with the ethical principles of the Declaration of Helsinki (2013), Good Clinical Practice (GCP) according to ICH (pharmaceuticals) as well as ISO14155:2020 (medical devices), and regulatory requirements of the Netherlands. Approvals of the ethics committee (Máxima Medisch Centrum, Veldhoven) and the Dutch competent authority (CCMO) were obtained on 25 May and 3 June 2021, respectively, prior to study start. The study adheres to the consolidated standards of reporting trials statement (CONSORT).

### Study population

Eligible for study participation were patients from the study sites’ patient population who had a slow-healing or nonhealing lower leg ulcer presumed to be caused by venous insufficiency. Refer to Table 1 for all inclusion and exclusion criteria. Note that after enrolment of 10 patients, the original criteria were amended to increase the recruitment rate. Informed consent was obtained from all participants.

**Table 1.** Original and amended inclusion and exclusion criteria

Original	Amended^a^
**Inclusion criteria**	
- Slow-healing or nonhealing lower leg ulcer presumed to be caused by venous insufficiency^b^ - Insufficient wound healing (defined as <30% surface area reduction) during the previous 2 weeks of standard wound care - Wound duration of at least 3 weeks - Minimum wound area of 1 cm^2^ and a maximum diameter of 3 cm - Ankle Brachial Pressure Index (ABPI) between 0.8 and 1.3^c^ - Minimum patient age of 18 years	- Slow-healing or nonhealing lower leg ulcer presumed to be caused by venous insufficiency^b^ - Insufficient wound healing (defined as <30% surface area reduction) during the previous 2 weeks of standard wound care - **[removed]** - Minimum wound area of **0.5** cm^2^ and a maximum diameter of **6** cm - Ankle Brachial Pressure Index (ABPI) between 0.8 and 1.3^c^ - Minimum patient age of 18 years
**Exclusion criteria**	
- Any known malignant wound degeneration - Treatment with systemic antibiotics - Treatment with immunosuppressive agents or oral corticosteroids, unless dose was stable for at least 2 months and did not exceed 7.5 mg/day prednisone or equivalent - Treatment with advanced wound therapies—such as negative pressure therapy, hyperbaric oxygen therapy, biologicals (*e.g.,* skin substitutes, growth factors), electrophysical therapy - Contraindications for direct-CAP treatment: ° Very exudative wound ° Implanted active electronic device, such as a pacemaker ° Electronic medical device attached to the body during treatment ° Metal implant in the wound area ° Conductive connection from outside to inside the body at or near the heart ° Epilepsy ° Pregnancy	- Any known malignant wound degeneration - Treatment with systemic antibiotics - Treatment with immunosuppressive agents or oral corticosteroids, unless dose was stable for at least 2 months and did not exceed 7.5 mg/day prednisone or equivalent - Treatment with advanced wound therapies—such as negative pressure therapy, hyperbaric oxygen therapy, biologicals (*e.g.,* skin substitutes, growth factors), electrophysical therapy - Contraindications for direct-CAP treatment: ° Very exudative wound ° Implanted active electronic device, such as a pacemaker ° Electronic medical device attached to the body during treatment ° Metal implant in the wound area ° Conductive connection from outside to inside the body at or near the heart ° Epilepsy ° Pregnancy
- Mixed etiology (venous and arterial) leg ulcer - Ulcer on the foot or the knee - Debridement intolerance - Compression therapy intolerance - Hospitalization at the time of inclusion or likely to occur in the near future - Vascular surgery related to the to-be-treated wound within the previous 2 months - Deep vein thrombosis in the previous 3 months - Uncontrolled diabetes mellitus (HbA1c >10%, 86 mmol/mol) within the previous 6 months; or unknown HbA1c - Comorbidity or other circumstance that is likely to compromise the outcome of the study or the feasibility of the patient fulfilling the study - COVID-19 infection in the previous 6 months with persistent symptoms	- **[removed]** - **[removed]** - **[removed]** - **[removed]** - **[removed]** - **[removed]** - **[removed]** - **[removed]** - **[removed]** - **[removed]**

^a^ Some criteria were removed or changed (indicated in bold) after enrolment of 10 patients (in January 2022), to increase the recruitment rate by simplifying the selection process and increasing the number of eligible patients.

^b^ There was no upper limit for the duration that the wound existed. In cases where a patient had multiple wounds that met the inclusion and exclusion criteria, the wound with the longest duration was identified for the study.

^c^ Patients with diabetes could be included based on a VLU diagnosis from anamnesis instead, since ABPI measurement is not always reliable for such patients.

### Randomization

Eligible participants were randomly allocated to one of three groups according to a randomization module present in the electronic data capture system, thus ensuring allocation concealment, with a 1:1:1 allocation using random block sizes of 3 and 6.

### Interventions

#### Standard of care

All three groups received SOC for 12 weeks or until healing, whichever occurred first. SOC was performed at the study site facilities, as well as at the patients’ residences by wound nurses, wound consultants, and nursing specialists. The treating health care professional determined the frequency of SOC for all study groups. SOC was the same for all groups and consisted of the study sites’ best practices, which are based on the Dutch guideline for VLUs,^13^ and include compression therapy and debridement. No restrictions, *e.g.,* on dressing types, were imposed specifically for this study. Debridement was required at least once per week (if clinically applicable). Sharp debridement was needed to be performed when necrotic tissue and/or callus was present in/around the wound. Details of the SOC provided were recorded during the treatment period and at the follow-up timepoints.

#### Direct-CAP treatment

In addition to SOC, the treatment groups received direct-CAP treatment according to the device’s instructions for use, either once or twice a week depending on group allocation, with at least one day between treatments.

The direct-CAP device (PLASOMA®, Plasmacure B.V., Nijmegen, The Netherlands) is a Class IIb CE-marked device. It consists of a power source and a flexible pad that is placed on the wound during the 2-minute treatment. The treatment program is automatically set and identical for all patients. The direct-CAP device is a volume dielectric barrier discharge (DBD) type of CAP device that turns the entire volume of air between pad and wound into CAP. Thus, the CAP makes direct contact with the wound, therefore also called ‘direct-CAP’, allowing all reactive species, including the short-living ones, and the electric field to have their effect on the wound. The CAP is created in a closed system, so that the reactive species cannot escape. Note that in this article, ‘direct-CAP’ always refers to the PLASOMA® device/treatment, whereas ‘CAP’ refers to cold atmospheric plasma in general.

### Outcomes and data collection

Patient demographics, medical history, current treatments, and wound characteristics were recorded at baseline. During the treatment period, study assessments were done once a week. Follow-up (FU) was performed at two timepoints: 2 weeks after end of treatment period (FU1) and 12 weeks after end of treatment period (FU2). Data were captured in a General Data Protection Regulation (GDPR) and GCP compliant web-based system.

Primary outcome was the percentage of wounds healed within 12 weeks treatment. Wound healing was defined as re-epithelialization without drainage or dressing requirements confirmed at two consecutive visits 2 weeks apart (FU1 was used to confirm healing), in line with recommendations from the U.S. Food & Drug Administration.^11^ Wound healing was based on the judgment of the treating health care professional; due to resource constraints, assessment of wound healing by independent blinded assessors was omitted.

Secondary outcomes included percentage wound area reduction, number of recurrences, and nature and incidence of adverse events (according to article 80 of the Medical Device Regulation).^14^ Data collected for other secondary outcomes have not been analyzed because of limited resources.

Note that initially, the treatment period lasted up to 20 weeks (the primary outcome timepoint was still 12 weeks). In May 2022, after enrolment of 22 patients and 6 subjects being past the 12-week timepoint, the treatment period was shortened to a maximum of 12 weeks to increase subject recruitment and limit dropouts. Two subjects received direct-CAP (once a week) for 3 additional weeks; the other 4 subjects were in the control group, receiving SOC either way. For these 6 subjects, follow-up data for a 12-week treatment period were obtained from treatment week 14 (FU1), and from the timepoint closest by (FU2).

Wound assessments were done after wound debridement (if applicable) and cleaning and for the treatment groups before direct-CAP treatment. The wound registration system inSight® (eKare, Nieuw-Vennep, The Netherlands) was used to determine wound area. When taking measurement photos with inSight® was impossible due to technical issues, photos with a ruler analyzed via ImageJ (version 1.53n 7 November 2021) were used to calculate wound area. Wound infection was graded according to the Society for Vascular Surgery Lower Extremity Threatened Limb (SVS WIfI) classification system.^15^

### Statistics

#### Sample size

The proportion expected to heal within 12 weeks for the control group was estimated to be 50%.^16–18^ Two scenarios for the percentage healing in the treatment group were taken into account. First, a relative difference of 25% is considered clinically relevant,^19^ *i.e.*, a treatment/control ratio of 1.25, leading to 62.5% healing in the treatment group. Second, in a clinical study on DFU, 55% seemed to have been converted from nonhealers to healers after two weeks direct-CAP treatment.^7^ In case of 50% healing in the control group, this would result in 77.5% healing in the treatment group (treatment/control ratio of 1.55). Based on 2-sided 95% confidence intervals (CIs) for both scenarios using a binomial distribution [R function BinomCI(), Wald CI], the sample size was set to 45 patients per group (50, including 10% dropouts, 150 patients in total), allowing for an indication of the difference (in case of scenario 1) and potentially a statistically significant difference in wound healing (in case of scenario 2) between control and treatment groups. Note that this sample size was not reached due to premature study termination.

#### Populations

All analyses were performed using the intention-to-treat (ITT) population, which included all randomized patients who met all study criteria prior to randomization and received at least one study treatment. The per-protocol (PP) population, which included all ITT patients who followed the protocol without significant deviations and did not withdraw or drop out, was used for sensitivity analyses. Excluded from the PP population were patients who received over 12 weeks direct-CAP treatment due to the initial 20-week treatment period.

#### Primary outcome analyses

The proportion of wounds healed with 95% CI for each group was estimated using Clopper–Pearson (Fisher’s exact) method. Clopper–Pearson CI is commonly used in calculating the exact CI for binomial proportion. The method is intended for the calculation of CI for a single group, not for calculating the CI for the difference between two groups. As a sensitivity analysis because of the relatively small sample size and highly conservative Fisher’s Exact method, the Wilson method was used, with 90% CIs of healing proportions calculated for each group. Wilson’s score CI is based on an asymmetrical distribution restricted to the probability range and allows to robustly depict uncertainty across all values of observed probability even with small sample size.^20^ The Wilson statistic without correction performs extremely well even compared with exact methods. In addition, healing proportions were compared using the Wang method (absolute and relative differences with 90% CIs) instead of with logistic regression, as originally planned, because of the smaller sample size. Wang’s method can be used to estimate the smallest CI for the difference of two proportions of two independent binomial random variables; it is constructed based on a direct analysis of coverage probability function.^21^ To show the probability that a wound is not healed, a *post hoc* nonparametric survival analysis using Kaplan–Meier curves was constructed.

#### Secondary outcomes analyses

For the secondary outcome wound area reduction, the analysis of covariance (ANCOVA) model was used, with logs of individual relative reductions as dependent variable, control and two treatment groups as fixed effect, and subjects nested within group as random effect. Wound area baseline was included as covariate; the presence of wound infection was not included as no wound infection was observed. The 95% CIs were constructed around the difference between least square means of treatment and control. Data were retransformed to the original scale to obtain a ratio of treatment versus control groups. As *post hoc* analyses, paired differences were calculated as decrease with respect to baseline and mean differences compared using paired two-sample Student’s *t-*test with unequal variances. In addition, mean wound area was calculated per group for each timepoint; for wounds healed earlier than after 12 weeks of treatment, last observation that carried forward imputation method was used. Descriptive statistics was calculated for ulcer recurrence, debridement frequency, and number of direct-CAP treatments until healing (calculated from number of weeks until healing; some direct-CAP treatments were missed, so actual number of treatments are lower).

#### General

Because of the exploratory nature of the study, no adjustment to control type 1 error was considered. A significance level of *p* < 0.05 was considered statistically significant. Statistical analyses were performed by an independent biostatistician using R software version 4.3.1 (R Foundation for Statistical Computing, Vienna, Austria, 2023).

## RESULTS

**Figure 2.**
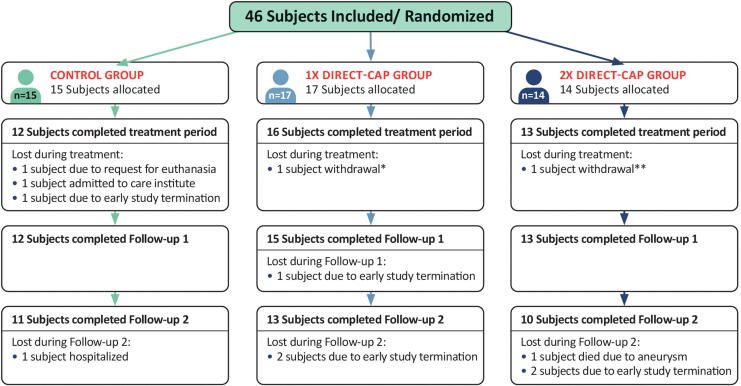
CONSORT flow diagram showing participant flow. All randomized subjects received the allocated treatment. No subjects were excluded from analysis for the ITT population. * Subject experienced a reaction in the leg (red/ thick/ warm); unclear if it was related to direct-CAP; this reaction was not seen/ could not be confirmed by the treating health care professional, who reported that it was not related to the study. ** Subject did not see enough wound healing progress and did not want to continue because of increasing pain complaints. Treating health care professional reported that it was not related to the study. CONSORT, consolidated standards of reporting trials; ITT, intention-to-treat.

Patients were enrolled between July 2021 and April 2023. Forty-six patients were randomly allocated to receive SOC (*n* = 15), SOC + direct-CAP once a week (*n* = 17), or SOC + direct-CAP twice a week (*n* = 14) (Fig. 2). The treatment period was completed by 12 out of 15 (80%) randomly assigned participants in the control group, 16 out of 17 (94%) in the 1× direct-CAP group, and 13 out of 14 (93%) in the 2× direct-CAP group. A total of 12 patients (26%) did not complete the treatment period or follow-up, of which 6 due to early study termination and 3 (control), 1 (1× direct-CAP), and 2 (2× direct-CAP) due to other reasons. The ITT analysis included all 46 patients. For the PP analysis (*n* = 26), 5 (30%) patients in the control group, 7 (41%) patients in the 1× direct-CAP group, and 8 (57%) patients in the 2× direct-CAP group were excluded because of significant protocol deviations or premature study discontinuation. The study was prematurely terminated due to slow recruitment and budget constraints, making continuation of the study infeasible for the sponsor.

### Baseline characteristics

Table 2 presents the demographics and baseline characteristics of the ITT population. Due to the lower number of subjects, the baseline characteristics of enrolled patients were not all well balanced between groups. Differences in the most important prognostic factors^22–24^ or noteworthy disparities between groups are mentioned. The 1× direct-CAP group had a higher prevalence of individuals with comorbidities, notably heart failure, than the other two groups, and the highest percentage of patients on comedication. The 2× direct-CAP group had shorter wound durations on average. The control group had more wounds on the foot and the smallest wounds on average; the higher mean wound size in the 1× direct-CAP group is mainly caused by one relatively large wound.

**Table 2.** Demographics and baseline characteristics of the ITT population

	Control (*n* = 15)	1× direct-CAP (*n* = 17)	2× direct-CAP (*n* = 14)
**Patient Characteristics**			
Gender			
Female	10 (67%)	11 (65%)	11 (79%)
Male	5 (33%)	6 (35%)	3 (21%)
Age (years)	77 (14)	75 (11)	75 (13)
White/Caucasian ethnicity	15 (100%)	17 (100%)	14 (100%)
BMI (kg/m^2^)	27.5 (6.2)	29.1 (5.6)	27.9 (6.4)
Comorbidity^a^	10 (67%)	14 (82%)	5 (36%)
Renal failure	1 (6.7%)	1 (5.9%)	2 (14%)
Heart failure	5 (33%)	10 (59%)	2 (14%)
Immobility	1 (6.7%)	1 (5.9%)	0 (0%)
Impaired vision	0 (0%)	2 (12%)	0 (0%)
Depression	0 (0%)	1 (5.9%)	1 (7.1%)
Dementia	0 (0%)	0 (0%)	0 (0%)
Other comorbidity—all	7 (47%)	5 (29%)	2 (14%)
Other comorbidity—hypertension	4 (57%)	2 (40%)	0 (0%)
Comedication	11 (73%)	17 (100%)	7 (50%)
Diabetes (all type II)	3 (20%)	3 (18%)	1 (7.1%)
Ankle-brachial pressure index	1.02 (0.16)	1.09 (0.13)	1.03 (0.15)
Number of wounds	1.27 (0.80)	1.59 (0.94)	1.14 (0.36)
History of previous ulceration at another location	9 (60%)	9 (53%)	6 (43%)
**Wound characteristics**			
Duration of wound (days)	188 (191)	181 (199)	115 (137)
*Minimum, Median, Maximum*	21, 98, 700	24, 90, 700	21, 70, 550
Wound area (cm^2^)	1.81 (1.68)	3.66 (4.17)	2.41 (1.36)
*Minimum, Median, Maximum*	0.40, 1.05, 6.10	0.70, 3.16, 18.10	0.50, 2.70, 4.49
Wound infection	0 (0%)	0 (0%)	0 (0%)
Clinical signs VLU			
C1 Telangiectasia or Reticular veins	6 (40%)	4 (24%)	2 (14%)
C2 Varicose veins	10 (67%)	8 (47%)	5 (36%)
C3 Edema	11 (73%)	15 (88%)	12 (86%)
C4a Pigmentation or eczema	7 (47%)	6 (35%)	4 (29%)
C4b Lipodermatosclerosis or Atrophie blanche	3 (20%)	2 (12%)	2 (14%)
C1-C4b: Venous insufficiency^b^	14 (93%)	17 (100%)	14 (100%)
C5 Healed venous ulcer	1 (6.7%)	3 (18%)	1 (7.1%)
C6 Active venous ulcer	15 (100%)	17 (100%)	14 (100%)
Wound classification 1			
A: Medial	11 (73%)	13 (76%)	6 (43%)
B: Not medial	4 (27%)	4 (24%)	8 (57%)
Wound classification 2			
C: Primary	7 (47%)	13 (76%)	10 (71%)
D: Recurrence	8 (53%)	4 (24%)	4 (29%)
Anatomical depth—superficial wound	15 (100%)	17 (100%)	14 (100%)
Necrotic tissue	1 (6.7%)	0 (0%)	0 (0%)
Peri-wound skin condition			
Healthy	9 (60%)	12 (71%)	12 (86%)
Erythematous	0 (0%)	0 (0%)	2 (14%)
Eczema	2 (13%)	2 (12%)	0 (0%)
Other	4 (27%)	3 (18%)	0 (0%)
Wound location			
Foot	9 (60%)	4 (24%)	5 (36%)
Shin	5 (33%)	12 (71%)	7 (50%)
Calf	1 (6.7%)	1 (5.9%)	2 (14%)

Data are means (SD) or numbers (%), unless otherwise specified. BMI, body mass index; ITT, intention-to-treat; VLU, venous leg ulcer.

^a^ Number of experienced cases (one subject can have more cases).

^b^ Venous insufficiency in case at least one of the clinical signs C1-C4b is present.

### Standard of care

SOC, as well as direct-CAP treatments and wound assessments, was performed at the study site in 55% and at home in 45% of the visits. Average frequency of SOC visits was approximately once a week for the control and 1× direct-CAP groups and closer to twice a week for the 2× direct-CAP group. The majority of the subjects in all three groups received therapeutic elastic compression stockings class 2 (30–40 mmHg). The mean debridement frequency [considering only wounds for which debridement was indicated (*n* = 28)] was more than once a week for all groups, which is considered frequent and shown to correlate with higher healing rates than debridement frequencies below once a week.^25^

### Primary outcome: Wound healing

**Figure 3.**
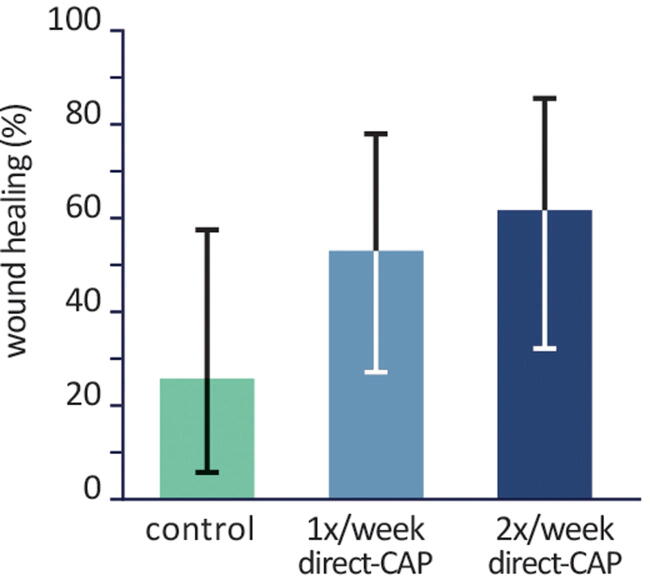
Percentages of wounds healed within 12 weeks (ITT population). Bars represent means, error bars show Fisher’s Exact 95% CIs. Differences are not statistically significant. CI, confidence interval; ITT, intention-to-treat.

A higher percentage of wounds healed within 12 weeks in the treatment groups: 53.3% (1× direct-CAP) and 61.5% (2× direct-CAP) versus 25.0% (control) (Fig. 3 and Table 3). Large differences between treatment groups and control were observed: absolute/relative difference 28.3%/113.3% (*p* = 0.16) for 1× direct-CAP and 36.5%/146.2% (*p* = 0.07) for 2× direct-CAP.

**Table 3.** Primary and secondary outcomes, sensitivity, and post hoc analyses

	Control	1× direct-CAP	2× direct-CAP
**Primary outcome—wound healing within 12 weeks**			
n/N (%)—ITT	3/12 (25.0%)	8/15 (53.3%)	8/13 (61.5%)
95% CI (Fisher’s exact)	5.5–57.2%	26.6 to 78.7%	31.6 to 86.1%
*90% CI* (Wilson)	*10.5–48.7%*	*33.3 to 72.3%*	*39.3 to 79.8%*
*n/N (%)—PP*	*3/10 (30.0%)*	*7/10 (70.0%)*	*3/6 (50.0%)*
*95% CI* (Fisher’s exact)	*6.7–65.3%*	*34.8 to 93.3%*	*11.8 to 88.2%*
*90% CI* (Wilson)	*12.7–55.8%*	*44.2 to 87.3%*	*22.1 to 77.9%*
Difference with control—ITT			
Absolute % / relative %	NA	28.3% / 113.3%	36.5% / 146.2%
90% CI (Wang)	NA	−4.7 to 56.9% / −18.8 to 227.6%	4.2 to 63.6% / 16.8 to 254.4%
*p* value	NA	0.1560	0.0746
*Difference with control—PP*			
*Absolute % / relative %*	*NA*	*40.0% / 133.3%*	*20.0% / 66.7%*
*90% CI (Wang)*	*NA*	*2.5 to 69.2 /* *8.3 to 230.7*	*−26.5 to 59.7 / −88.3 to 199.0*
*p* value	*NA*	*0.0800*	*0.4955*
**Secondary outcome—wound area reduction**			
Difference with control within 12 weeks—ITT			
LSmean (SE)	NA	11.9% (22.3%)	46.0% (22.6%)
95% CI	NA	−33.3 to 57.1%	0.1 to 92.0%
*p* value	NA	0.5782	0.0717
Difference with control at FU1—ITT			
LSmean (SE)	NA	20.8% (13.0%)	38.8% (12.8%)
95% CI	NA	−5.5 to 47.1%	12.9 to 64.7%
*p* value	NA	0.1305	0.0100
Difference between wound area at 12 weeks and baseline—ITT (cm^2^)*			
Mean (SD)	1.15 (2.03)	1.97 (1.99)	2.37 (1.27)
95% CI	−0.30 to 2.60	0.91 to 3.03	1.60 to 3.14
*p* value	0.1073	0.00128	0.00002

Sensitivity analyses: in italics. Post hoc analysis: indicated with asterisk.

CI, confidence interval; FU, follow-up; ITT, intention-to-treat; NA, not applicable; PP, per-protocol; SD, standard deviation; SE, standard error.

The results of sensitivity analyses (Table 3) were generally in agreement with those of the primary analysis. The only clear discrepancy is the group with the highest percentage wound healing: in the ITT population, this was the 2× direct-CAP group, whereas in the PP population, this was the 1× direct-CAP group. This inconsistency is most likely caused by the low number of wounds assessed for the PP population, especially for the 2× direct-CAP group. The results of all analyses are shown in Table 3.

**Figure 4.**
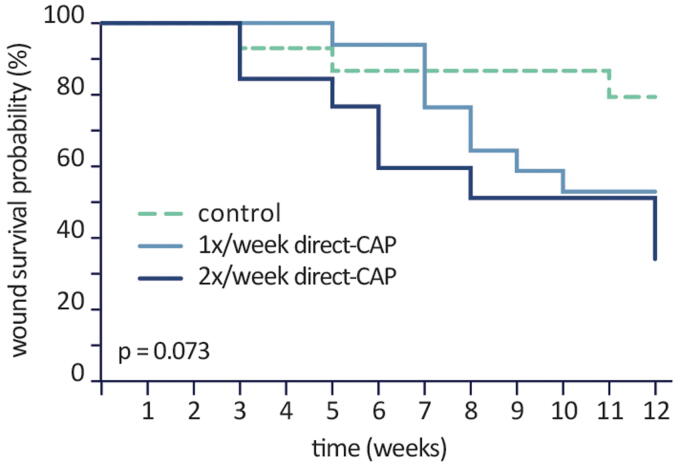
Kaplan–Meier curve (ITT population). Probability that a wound is not healed (wound survival) during the treatment period. Differences are not statistically significant.

As a *post hoc* sensitivity analysis, Kaplan–Meier curves show the probability that a wound is not healed (wound survival) during the treatment period; the lower the wound survival probability, the better the treatment efficacy (Fig. 4). In the control group the probability of wound survival remains at 79% after 12 weeks of treatment, contrary to the treatment groups, which show a wound survival of 53% (1× direct-CAP) and 34% (2× direct-CAP) (*p* = 0.07).

To further compare the two treatment groups, the number of treatments until wound healing was calculated. The 2× direct-CAP group [mean 13.8, standard deviation (SD) 7.1, range 6–24] received on average 1.8 times more treatments until their wounds healed compared with the 1× direct-CAP group (mean 7.6, SD 1.5, range 5–10).

### Secondary outcomes

#### Wound area reduction

**Figure 5.**
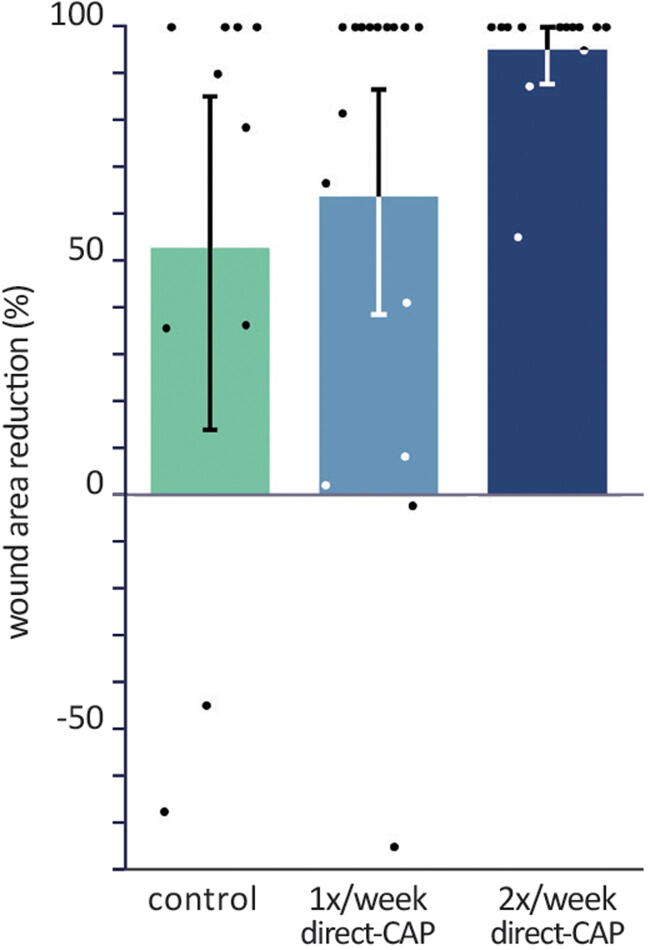
Percentages of wound area reduction within 12 weeks (ITT population). Bars represent means, error bars show 95% CIs, and dots show single data points. Differences are not statistically significant.

The largest wound area reduction within 12 weeks was obtained in the 2× direct-CAP group (95.2%), followed by 63.9% (1× direct-CAP) and 52.8% (control) (Fig. 5 and Table 3). Despite the nearly 100% wound area reduction and large absolute difference compared with control (46.0%), it cannot be declared as statistically significant (*p* = 0.07), which is confirmed by the ANCOVA results (Table 4). The factor of baseline appeared to be insignificant (*p* = 0.95). Noteworthy is that the mean wound area reduction at FU1 significantly differed between the three groups (*p* = 0.03); the largest difference was found between 2× direct-CAP and control (38.8%, *p* = 0.01).

**Table 4.** Secondary outcome—wound area reduction—ANCOVA (ITT)

Source of variability	df	Mean Square	F Value	Pr(>F)
Within 12 weeks				
Treatment groups	2	0.4533	1.9487	0.1576
Baseline area	1	0.0008	0.0033	0.9545
Residuals	35	0.2326		
At FU1				
Treatment groups	2	0.2945	3.7234	0.0345
Baseline area	1	0.0259	0.3273	0.5710
Residuals	34	0.0791		

ANCOVA, analysis of covariance; FU, follow-up; ITT, intention-to-treat.

**Figure 6.**
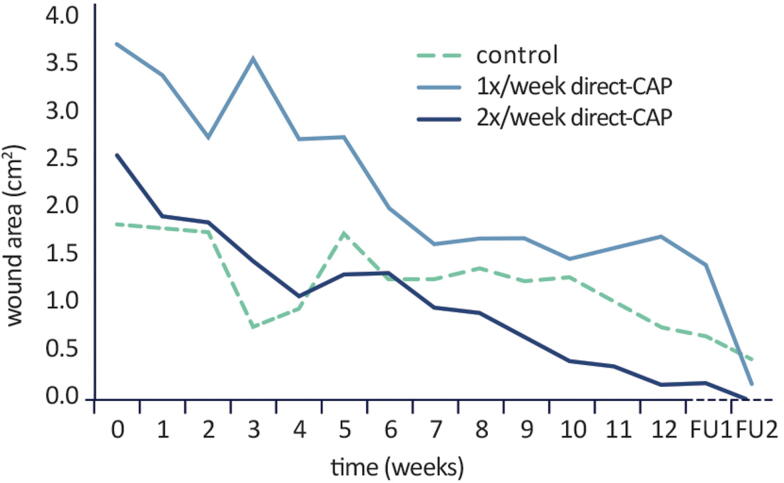
Course of mean wound area within treatment period and at subsequent follow-up timepoints (after 2 weeks, FU1, and 12 weeks, FU2) (ITT population). No statistical test performed.

As *post hoc* analysis, absolute reductions in wound area at 12 weeks with respect to baseline were calculated; a significant decrease was observed for both treatment groups (*p* = 0.001 for 1× direct-CAP, *p* = 0.00002 for 2× direct-CAP), but not for control (*p* = 0.11). Furthermore, the course of the mean wound area is visualized (Fig. 6). Both treatment groups exhibited a relatively stable decrease of wound area during the treatment and FU periods, in contrast to the control group. The mean wound area in the 1× direct-CAP group remained above the control value until FU1 due to the higher baseline value.

#### Recurrence rate

Of the wounds that were healed at FU1, only 1 reopened (after local trauma) within the follow-up period, resulting in a recurrence rate of 12.5% in the 2× direct-CAP group and 0% in the other two groups.

#### Serious adverse events

Three serious adverse events (SAEs) were reported: in the control group, one patient was admitted to hospital (reason unknown) and another to a care institute (after a fall), and in the 2× direct-CAP group, one patient died due to an aneurysm. All three SAEs were not anticipated and not related to direct-CAP treatment.

### Acceptability direct-CAP

Overall, the health care providers found the direct-CAP device easy to use and both users and patients were satisfied with the treatments.

## DISCUSSION

This RCT shows the effect of direct-CAP on healing of chronic VLUs: 62% complete wound closure with 2× direct-CAP treatment a week and 53% with 1× direct-CAP treatment a week versus 25% with SOC only and an impressive wound area reduction of 95% with 2× direct-CAP treatment a week. These results align with the existing clinical data on the direct-CAP device,^7–9^ further supporting its potential to improve the healing process of complex wounds and thus enhance patient outcomes.

### First RCT on CAP on complete wound closure

This is the first RCT that primarily investigates the effect of CAP on incidence of complete wound closure, one of the most objective and clinically meaningful wound healing outcomes,^11^ and with a sufficiently long treatment period of 12 weeks. Most CAP studies assess nonhealing outcomes, either intermediate or surrogate. Only one RCT included complete wound closure, merely as secondary outcome,^26^ and only one RCT had an intervention period of 12 weeks.^12^

### First RCT on CAP in home care

Direct-CAP treatments were performed by home care organizations both in specialized wound care facilities and within the patients’ residences. Eighty percent of wound care patients are treated at home and the majority of advanced treatments, including certain CAP treatments, can currently only be applied in health care facilities.^27^ None of the existing RCTs on CAP treatment for chronic wound healing has been executed outside health care facilities.

### Both treatment frequencies seem effective

The effect on wound healing may be similar for both treatment frequencies, considering that patients in the 1× direct-CAP group generally had wounds at higher risk of impaired healing, which may (partially) explain the lower healing rate compared with 2× direct-CAP. Nonetheless, the average number of treatments until healing in the 2× direct-CAP group was nearly twice the number in the 1× direct-CAP group. Note that the treatment duration was maximized at 12 weeks; treating all wounds until healing may alter these numbers.

One other study comparing CAP treatment frequencies has been published, which showed that once weekly CAP treatment was not inferior to CAP treatment thrice weekly.^12^

In conclusion, direct-CAP treatment once a week appears to be sufficient to achieve excellent results and is advisable from cost-benefit and practical perspectives. Temporarily higher treatment frequency may be beneficial in certain wounds, depending on the stage of wound healing and presence of wound infection.

### Efficacy in other complex wound types

Leg ulcers with venous and mixed etiology were included in the study. The observed effects of direct-CAP treatment are expected to also occur in other wound types, since the main causative factors of chronic wound pathogenesis are irrespective of the wound’s origin^28^ and can be diminished by CAP treatment.^6^ This is in line with clinical data on direct-CAP and other CAP devices in various complex wound types.^5–9^

### Limitations of the study

The main limitations of this study are the premature termination, exploratory nature, and open-label design.

#### Premature study termination and exploratory nature

Enrolment started mid-2021, a period significantly influenced by the COVID-19 pandemic. This had a major and enduring impact on the number of new VLU patients presenting at the study sites: Zuyderland Thuiszorg experienced a marked reduction, from 980 VLU patients treated in 2019 to 113 in 2020 (11.5% of 2019). This reduction surpasses what is reported in a National Health Service study, where the VLU incidence in 2022 was 22.6% of the 2019 figures.^29^ Due to the low recruitment rate, our study had to be terminated prematurely. Despite the smaller sample size and thus larger CIs, which limit the statistical significance of the results, this study clearly indicates the efficacy of direct-CAP treatment with impressive healing rates compared with SOC.

Because of the exploratory nature of the study, no control of type 1 error was considered, and thus, *p*-values are only for indicative purposes.

#### Open-label design

Blinding subjects and care providers was not feasible, which may have introduced a placebo effect or affected compliance. Nevertheless, RCTs with other CAP devices have demonstrated beneficial effects on wound healing compared with placebo.^12,30,31^ Furthermore, placebo effects were shown not to affect the objectively assessable outcomes of wound healing and wound size reduction.^32,33^ In the current study, control and 1× direct-CAP groups showed similar numbers of missed treatments with and without valid reason, while in the 2× direct-CAP group these numbers were higher (also when compensating for the higher treatment frequency); thus, compliance appears to be lower.

Blinded data assessment by independent assessors could not be performed as initially planned. Consequently, wound healing was assessed by the treating health care professional, which may have caused detection bias. However, wound healing was precisely defined and is generally not debatable.

#### Other

Random and concealed allocation were performed to avoid selection bias. However, the lower sample size led to more disparity in baseline characteristics, with the 1× direct-CAP group apparently being at a disadvantage. Dropout rates were similar in the three groups and causes did not systematically differ; thus, attrition bias did not occur. Adjustment of the treatment period duration impacted only the follow-up data for 2 subjects, in the 1× direct-CAP group.

A single- or double-blinded RCT or open-label RCT with blinded data assessment for the primary endpoint, as well as a larger study population, is desired to confirm the presented results.

### Conclusions

The results of this RCT indicate that direct-CAP treatment can substantially improve wound healing outcomes of slow-healing and nonhealing VLUs compared with SOC alone. Importantly, this is the first RCT showing that CAP is effective in primary care settings. Direct-CAP treatment once a week appears to be sufficient and is advisable from cost-benefit and practical perspectives. Together with safety and efficacy data from other clinical studies, these results support the integration of direct-CAP as a valuable and user- and patient-friendly therapeutic option in the management of complex wounds.

## ABOUT THE AUTHORS

**Olaf Bakker, MD, PhD,** is a vascular surgeon in the St. Antonius hospital and is specialized in advanced endovascular therapies, as well as limb salvage procedures, with a special interest in wound healing in patients with diabetes. **Paulien Smits, PhD,** is a biomedical scientist at Plasmacure B.V. and involved in clinical studies with PLASOMA. **Chantal van Weersch, MSc,** is a wound consultant and nurse practitioner at Zuyderland home care in a nurse-led team. She is focused on complex wound care and dermatology. **Melissa Quaaden, MSc,** is a wound consultant and nurse practitioner at Zuyderland home care in a nurse-led team. She is focused on complex wound care and vascular pathology. **Esther Bruls, MSc,** is a wound consultant and nurse practitioner at Zuyderland home care in a nurse-led team. She is focused on complex wound care and diabetic foot ulcers. **Angela van Loon, MSc,** is a wound nurse and advanced nurse practitioner specialized in complex wound treatment at Thebe home care (WondExpertiseCentrum West and Central Brabant) and the Elisabeth-TweeSteden hospital. **Joost van der Kleij, MD,** is a dermatologist with lifelong experience in wound healing, more specific venous leg ulcers and diabetic feet, and initiator of multidisciplinary wound teams in the hospitals he worked in, currently working as independent consultant.

## Abbreviations and Acronyms

ABPI: ankle brachial pressure index
ANCOVA: analysis of covariance
CAP: cold atmospheric plasma
CI: confidence interval
CONSORT: consolidated standards of reporting trials
Direct-CAP: direct cold atmospheric plasma
DFU: diabetic foot ulcer
FU: follow-up
GCP: good clinical practice
ITT: intention-to-treat
PP: per-protocol
RCT: randomized controlled trial
SAE: serious adverse event
SD: standard deviation
SOC: standard of care
VLU: venous leg ulcer

## References

[1] Probst S, Saini C, Gschwind G, et al. Prevalence and incidence of venous leg ulcers-A systematic review and meta-analysis. Int Wound J 2023;20(9):3906–3921; doi: 10.1111/iwj.14272 37293810 PMC10588327

[2] Falanga V, Isseroff RR, Soulika AM, et al. Chronic wounds primer. Nat Rev Dis Primers 2022;8(1):50; doi: 10.1038/s41572-022-00377-3 35864102 PMC10352385

[3] Kolimi P, Narala S, Nyavanandi D, et al. Innovative treatment strategies to accelerate wound healing: Trajectory and recent advancements. Cells 2022;11(15):2439; doi: 10.3390/cells11152439 35954282 PMC9367945

[4] Frykberg RG, Banks J. Challenges in the treatment of chronic wounds. Adv Wound Care (New Rochelle) 2015;4(9):560–582; doi: 10.1089/wound.2015.0635 26339534 PMC4528992

[5] Wiegand C. Potential of Cold Atmospheric Pressure Plasma (CAPP) in wound management. Wounds Int 2019;10(4):26–31.

[6] Bekeschus S, von Woedtke T, Emmert S, et al. Medical gas plasma-stimulated wound healing: Evidence and mechanisms. Redox Biol 2021;46:102116; doi: 10.1016/j.redox.2021.102116 34474394 PMC8408623

[7] Lagrand RS, Sabelis LW, de Groot V, et al. Cold plasma treatment is safe for diabetic foot ulcers and decreases staphylococcus aureus bacterial load. J Wound Care 2023;32(4):247–251; doi: 10.12968/jowc.2023.32.4.247 37029969

[8] Lim K, Hieltjes M, van Eyssen A, et al. Cold plasma treatment. J Wound Care 2021;30(9):680–683; doi: 10.12968/jowc.2021.30.9.680 34554832

[9] Stoeldraaijers L, Spencer GJ, Perez AZ, et al (3 May 2023). Cold plasma treatment for chronic wounds in a low-income country: A case series. [Poster Presentation] EWMA : Milan, Italy.

[10] Piaggesi A, Låuchli S, Bassetto F, et al. Advanced therapies in wound management: Cell and tissue based therapies, physical and bio-physical therapies smart and IT based technologies. J Wound Care 2018;27(Sup6a):S1–S137; doi: 10.12968/jowc.2018.27.Sup6a.S1 29902114

[11] U.S. Department of Health and Human Services Food and Drug Administration (FDA). Guidance for industry: Chronic cutaneous ulcer and burn wounds - developing products for treatment. 2006.

[12] Moelleken M, Jockenhöfer F, Wiegand C, et al. Pilot study on the influence of cold atmospheric plasma on bacterial contamination and healing tendency of chronic wounds. J Dtsch Dermatol Ges 2020;18(10):1094–1101; doi: 10.1111/ddg.14294 32989866

[13] Nederlandse Vereniging voor Dermatologie en Venereologie (NVDV). Richtlijn Diagnostiek En Behandeling van Het Ulcus Cruris Venosum. 2005.

[14] Official Journal of the European Union. Regulation (EU) 2017/745 of the European parliament and of the council of 5 April 2017 on medical devices. 2017.

[15] Mills JL, Conte MS, Armstrong DG, et al. The society for vascular surgery lower extremity threatened limb classification system: Risk stratification based on Wound, Ischemia, and Foot Infection (WIfI). J Vasc Surg 2014;59(1):220–234.e2; doi: 10.1016/j.jvs.2013.08.003 24126108

[16] Nelson EA, Adderley U. Venous leg ulcers. BMJ Clin Evid 2016;2016:1902. PMC4714578 26771825

[17] Guest JF, Fuller GW, Vowden P. Venous leg ulcer management in clinical practice in the UK: Costs and outcomes. Int Wound J 2017;15(1):29–37; doi: 10.1111/iwj.12814 29243398 PMC7950152

[18] Nicolaides AN. Investigation of chronic venous insufficiency. Circulation 2000;102(20):e126–e163; doi: 10.1161/01.CIR.102.20.e126 11076834

[19] Nederlandse Internisten Vereniging (NIV). Richtlijn Diabetische Voet; 2017. Available from: https://richtlijnendatabase.nl/richtlijn/diabetische_voet/startpagina_diabetische_voet.html [Last accessed: October 25, 2023].

[20] Wallis S. Binomial confidence intervals and contingency tests: Mathematical fundamentals and the evaluation of alternative methods. J Quant Linguist 2013;20(3):178–208; doi: 10.1080/09296174.2013.799918

[21] Wang W. On construction of the smallest one-sided confidence interval for the difference of two proportions. Ann Statist 2010;38(2):1227–1243; doi: 10.1214/09-AOS744

[22] Velickovic VM, Macmillan T, Kottner J, et al. Prognostic models for clinical outcomes in patients with venous leg ulcers - A systematic review. J Vasc Surg Venous Lymphat Disord 2023;12(1):101673; doi: 10.1016/j.jvsv.2023.06.017 37689364 PMC11523447

[23] Cho SK, Mattke S, Gordon H, et al. Development of a model to predict healing of chronic wounds within 12 weeks. Adv Wound Care 2020;9(9):516–524; doi: 10.1089/wound.2019.1091 PMC7522633 32941121

[24] Berezo M, Budman J, Deutscher D, et al. Predicting chronic wound healing time using machine learning. Adv Wound Care 2022;11(6):281–296; doi: 10.1089/wound.2021.0073

[25] Wilcox JR, Carter MJ, Covington S. Frequency of debridements and time to heal: A Retrospective Cohort Study of 312 744 wounds. JAMA Dermatol 2013;149(9):1050–1058; doi: 10.1001/jamadermatol.2013.4960 23884238

[26] Strohal R, Dietrich S, Mittlböck M, et al. Chronic wounds treated with cold atmospheric plasmajet versus best practice wound dressings: A multicenter, randomized, non-inferiority trial. Sci Rep 2022;12(1):3645; doi: 10.1038/s41598-022-07333-x 35256635 PMC8901692

[27] Capgemini Consulting. Innovatie van Complexe Wondzorg - Onderzoek Naar Potentiële Besparingen En Prestatieomschrijvingen —. In: Opdracht Van de Nederlandse Zorgautoriteit 2014

[28] Mustoe TA, O’Shaughnessy K, Kloeters O. Chronic wound pathogenesis and current treatment strategies: A unifying hypothesis. Plast Reconstr Surg 2006;117(7 Suppl):35S–41S; doi: 10.1097/01.prs.0000225431.63010.1b 16799373

[29] Guest JF, Fuller GW. Cohort study assessing the impact of COVID-19 on venous leg ulcer management and associated clinical outcomes in clinical practice in the UK. BMJ Open 2023;13(2):e068845; doi: 10.1136/bmjopen-2022-068845 PMC9944296 36806131

[30] Stratmann B, Costea T-C, Nolte C, et al. Effect of cold atmospheric plasma therapy vs standard therapy placebo on wound healing in patients with diabetic foot ulcers: A randomized clinical trial. JAMA Netw Open 2020;3(7):e2010411; doi: 10.1001/jamanetworkopen.2020.10411 32672829 PMC7366186

[31] Heinlin J, Zimmermann JL, Zeman F, et al. Randomized placebo-controlled human pilot study of cold atmospheric argon plasma on skin graft donor sites. Wound Repair Regen 2013;21(6):800–807; doi: 10.1111/wrr.12078 23937657

[32] Jockenhöfer F, Knust C, Benson S, et al. Influence of placebo effects on quality of life and wound healing in patients with chronic venous leg ulcers. J Dtsch Dermatol Ges 2020;18(2):103–109; doi: 10.1111/ddg.13996 31814307

[33] Mathur A, Jarrett P, Broadbent E, et al. Open-label placebos for wound healing: A randomized controlled trial. Ann Behav Med 2018;52(10):902–908; doi: 10.1093/abm/kax057 30212845

